# Utilization of machine learning methods for predicting surgical outcomes after total knee arthroplasty

**DOI:** 10.1371/journal.pone.0263897

**Published:** 2022-03-22

**Authors:** Hina Mohammed, Yihe Huang, Stavros Memtsoudis, Michael Parks, Yuxiao Huang, Yan Ma

**Affiliations:** 1 Milken Institute School of Public Health, The George Washington University, Washington, DC, United States of America; 2 Syapse Inc, Palo Alto, California, United States of America; 3 Hospital for Special Surgery, New York City, New York, United States of America; 4 Weill Cornell Medical College, Cornell University, New York City, New York, United States of America; 5 Columbian College of Arts & Sciences, The George Washington University, Washington, DC, United States of America; Assiut University Faculty of Medicine, EGYPT

## Abstract

**Background:**

Predictive models could help clinicians identify risk factors that cause adverse events after total knee arthroplasty (TKA), allowing for appropriate preoperative preventive interventions and allocation of resources.

**Methods:**

The National Inpatient Sample datasets from 2010–2014 were used to build Logistic Regression (LR), Gradient Boosting Method (GBM), Random Forest (RF), and Artificial Neural Network (ANN) predictive models for three clinically relevant outcomes after TKA—disposition at discharge, any post-surgical complications, and blood transfusion. Model performance was evaluated using the Brier scores as calibration measures, and area under the ROC curve (AUC) and F1 scores as discrimination measures.

**Results:**

GBM-based predictive models were observed to have better calibration and discrimination than the other models; thus, indicating comparatively better overall performance. The Brier scores for GBM models predicting the outcomes under investigation ranged from 0.09–0.14, AUCs ranged from 79–87%, and F1-scores ranged from 41–73%. Variable importance analysis for GBM models revealed that admission month, patient location, and patient’s income level were significant predictors for all the outcomes. Additionally, any post-surgical complications and blood transfusions were significantly predicted by deficiency anemias, and discharge disposition by length of stay and age groups. Notably, any post-surgical complications were also significantly predicted by the patient undergoing blood transfusion.

**Conclusions:**

The predictive abilities of the ML models were successfully demonstrated using data from the National Inpatient Sample (NIS), indicating a wide range of clinical applications for obtaining accurate prognoses of complications following orthopedic surgical procedures.

## Introduction

The American Joint Replacement Registry (AJRR) recorded that approximately one million total knee arthroplasty (TKA) procedures were conducted in the United States in 2020 [1], and this is further projected to increase to 1.5 million cases per year by 2050 [2]. Commonly cited complications after TKA are blood loss requiring blood transfusion [3], venous thromboembolisms [4], infections [5], and extended care upon discharge [6]. Predictive models could help clinicians identify risk factors that cause adverse events after surgery, allowing for appropriate preoperative preventive interventions and allocation of resources. For instance, TKAs have commonly been associated with high rates of blood transfusion [7] and the prediction of this complication prior to the surgery can help the clinicians predetermine the patient’s need for this limited resource, which can then be allocated accordingly. Logistic regression (LR) methods have commonly been used for predicting outcomes after TKA, such as length of stay [8], mortality [9], persistent pain [10] etc. The primary drawback for these regression models is that they require the users to specify the relationship between outcome and predictor variables, and incorrect specification leads to suboptimal predictive performance due to biased regression coefficients and invalid statistical inferences [11]. This issue is exaggerated while utilizing large administrative databases. With dozens or even hundreds of variables, as is often the case in such databases, model specification becomes a daunting task with no guarantee of success. Machine learning (ML) based predictive models have recently been increasingly explored in the field of orthopedics as powerful tool that can be utilized for conducting risk assessments in a clinical setting, as opposed to predictive models based on traditional regression methods. Unlike LR, ML automates analytical model building during data analysis by using algorithms that iteratively learn from data, and detect patterns, rules and statistical dependencies without being explicitly programmed where to look.

For the purposes of our study, we used multi-year data from the Healthcare Cost and Utilization Project National Inpatient Sample (HCUP NIS), the largest annual all payer database in the nation and sought to build predictive models using LR and ML methods for a set of clinically relevant surgical outcomes after TKA. ML is data driven and the more data fed into a ML system, the more it can learn and apply the results to higher quality prediction. Data scarcity is often responsible for poor performances in ML studies. Therefore, ML based predictive models built using large datasets, such as the NIS, are more likely to have accurate and dependable performance due to the large sample size of the study dataset. The outcomes under consideration in our study were–disposition of the patient at discharge, any post-surgical complications, and blood transfusion. After generating the various predictive models, the performance of these models were then compared and we sought to use the model with the best performance in order to generate patient-level predictions and better understand the relative relevance of the various predictor variables in influencing the likelihood of patients experiencing adverse outcomes after TKA.

## Methods

### Data source

This is a retrospective study (Level 3 evidence), conducted using the 2010–2014 NIS data. NIS annual data files are sponsored by the Agency of Healthcare Research and Quality. Detailed information on the NIS design can be found on the HCUP website [12]. Because the HCUP-NIS database includes only de-identified patient data, this study was deemed exempt by the George Washington University Committee on Human Research, Institutional Review Boards.

We identified patients who had undergone primary TKA between 2010–2014 as recorded in the NIS using the International Classification of Diseases, 9th Revision (ICD-9) procedure code, 81.54. Our outcomes of interest were disposition of patients at discharge, any post-surgical complications, and blood transfusion. Disposition of patients at discharge was categorized as either routine (patient’s residence) or non-routine (transfer to short-term, skilled nursing facility, intermediate care facility, another type of specialized care facility, home health care, against medical advice, death, and discharge alive with unknown destination). Blood transfusion was identified using the relevant ICD-9 procedure codes for blood transfusion and classified as “Received blood transfusion” or “Did not receive blood transfusion” (Table I, Appendix in S1 File). The outcome of any post-surgical complications was defined using a number of ICD-9 diagnosis codes for specific complications that occur after TKA (Table II, Appendix in S1 File), and was dichotomized as being either present or absent.

Predictors, including age, gender, race, admission month, admission on a weekend, admission type, health insurance, median household income for patient’s zip code, patient geographical location, ownership of hospital, bed size of hospital, location and teaching status of hospital, and comorbidities, namely—weight loss, valvular disease, solid tumor, renal failure, pulmonary circulation disorders, psychoses, peripheral vascular disorders, paralysis, obesity, other neurological disorders, fluid and electrolyte disorders, lymphoma, liver disease, hypothyroidism, hypertension, drug abuse, diabetes with chronic complications, depression, coagulopathy, chronic pulmonary disease, congestive heart failure, chronic blood loss anemia, rheumatoid arthritis, deficiency anemias and alcohol abuse, were used to predict the three outcomes of interest among patients who had a TKA.

### Statistical analysis

#### Model development and validation

**Fig 1:**
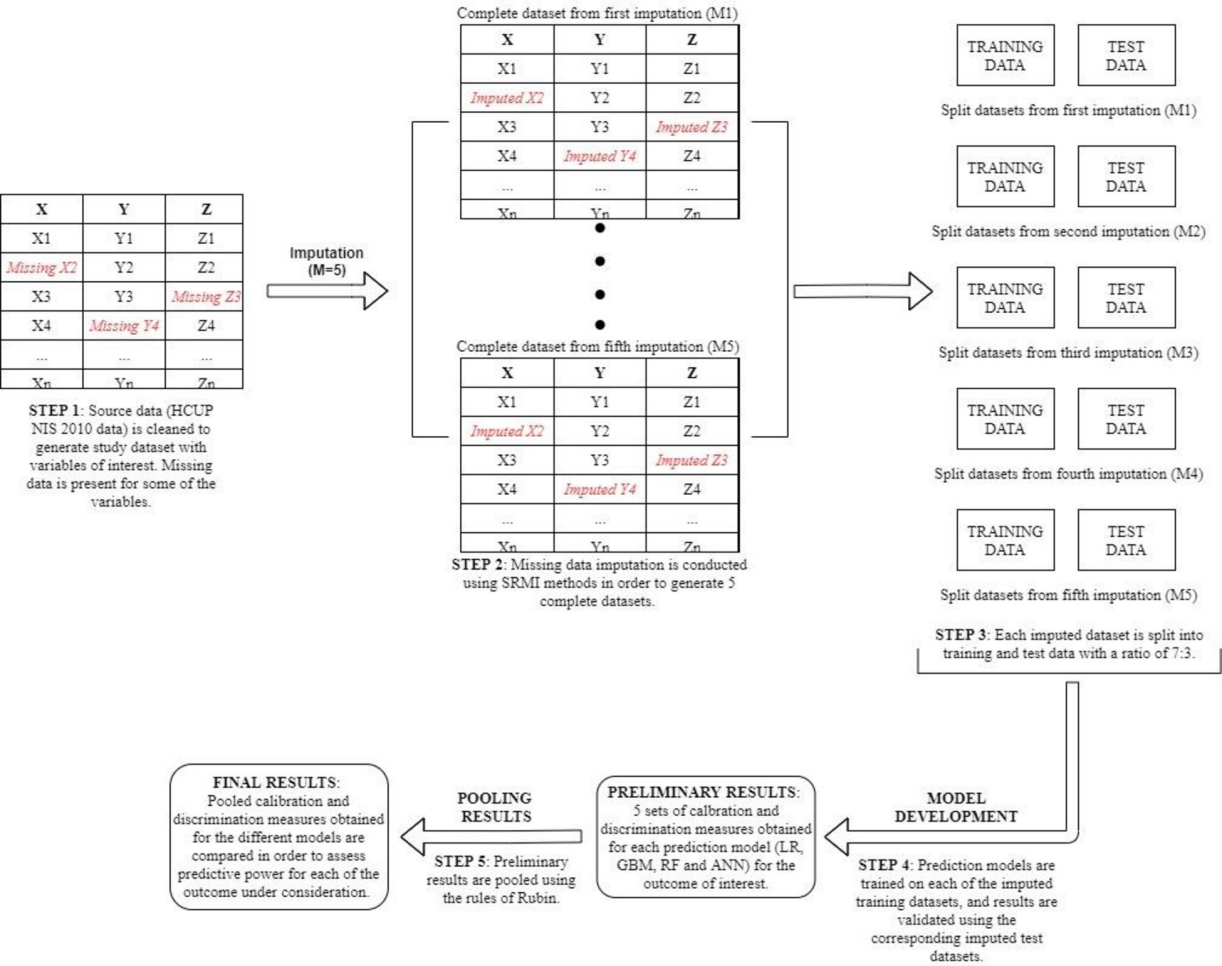
An illustrative flowchart of the development and validation of predictive models.

We developed predictive models based on logistic regression (LR) and three ML methods including gradient boosting machine (GBM), random forest (RF), and artificial neural networks (ANN). We deployed these three powerful methods inspired by the latest trends in ML. The description of the ML models, along with an outline of the parameter values used for these models, have been elaborated upon in the Appendix (S1–S3 Figs). Fig 1 consists of an illustrative flowchart that outlines the steps taken during model development for these predictive algorithms, for each of the outcomes of interest. In Step 1, we identified missing observations. In Step 2, missing data imputation was conducted using sequential regression multiple imputation (SRMI) [13,14] for handling the moderate amount of missing values for several predictors—such as race, admission month, and patient’s median household income for patient’s zip code. More specifically, missing data imputation was conducted for this study since in a recent study [15], we identified serious consequences (e.g., biased results) of ignoring missing data in the HCUP NIS and illustrated the advantage of advanced statistical methods such as multiple imputation (MI) for handling missing data. Step 2 resulted in 5 unique complete study datasets with no missing data. Given the large sample size of the NIS, 5 imputations should be sufficient [17]. In order to facilitate internal validation of the study results, each of these imputed datasets was then split into a training, validating, and test dataset [16] with a split ratio of 5:2:3 in Step 3. In the next step, predictive models were developed using each of the imputed training and validating datasets, and model prediction was conducted using the corresponding imputed test datasets. The performance of the predictive models was assessed using various calibration and discrimination measures, and the 5 sets of results from the imputed training and test datasets were pooled using the rules of Rubin [17] in Step 5 of the model development and validation process. Finally, the results were compared between the LR and ML based predictive models for the outcomes of interest.

As previously discussed, performances of the predictive models were evaluated based on two main measures, calibration and discrimination [18]. Calibration is a measure of the agreement between predicted probabilities and observed outcomes, and the measure utilized in this study was the Brier score, where a lower score is indicative of better model performance [19]. Discrimination refers to a model’s ability to discriminate between cases and non-cases; and that is usually measured the area under the receiver operating characteristic (ROC) curve (AUC), sensitivity, specificity and the F1-scores [20,21]. The sensitivity of a model is the measure of its ability to accurately predict positive outcomes, whereas the specificity of a model measures the ability to accurately predict negative outcomes. The AUC is a function of the sensitivity and specificity of a model, and its value ranges from 0 to 1. The F1-score for a model is a function of the positive predictive value (PPV), which is the percentage of positive outcomes that were accurately predicted by the model, and sensitivity of the model. AUC and F1-scores for the positive outcomes are reported in this study. Unlike the Brier score, the higher the F1-score and AUC, the better the model.

## Results

### Patient and hospital characteristics

**Fig 2:**
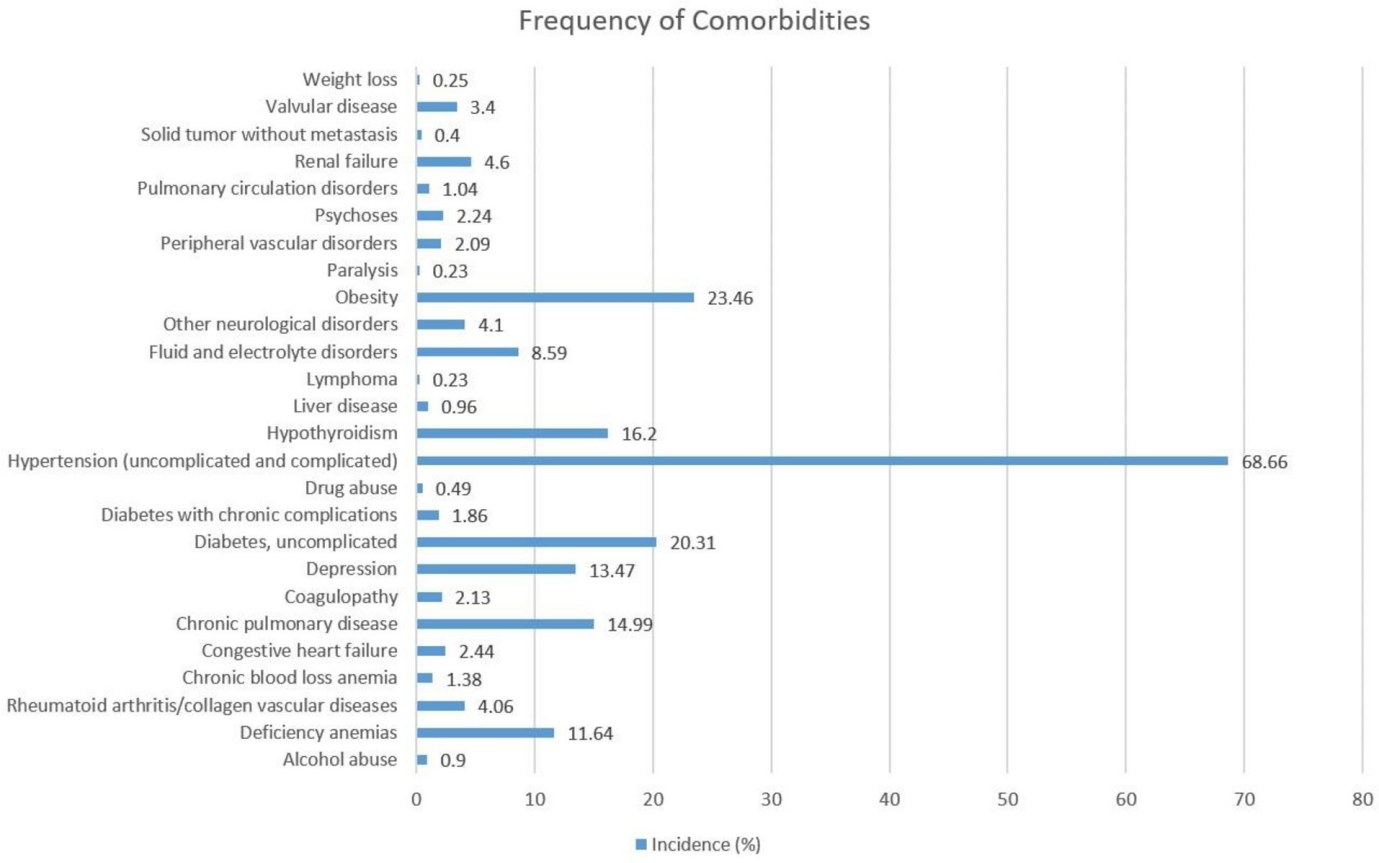
Frequency of comorbidities in the 2010–2014 NIS study dataset.

A total of 636,062 patients had undergone TKA between 2010 to 2014. These patients were primarily white (75.44%), female (62.33%), and between the ages of 44–64 (42.34%). A significant proportion of these patients had their healthcare costs covered by Medicare (55.52%) and private insurance (37.94%). Some of the comorbidities had a comparatively high prevalence in this population: hypothyroidism (16.2%), uncomplicated diabetes (20.31%), obesity (23.46%), and hypertension (68.66%), and the remaining comorbidities had a much lower prevalence in the study population (Fig 2). Other characteristics are outlined in Table 1, along with information of missing data in the original data. Race was found to have the highest degree of missingness, with 13.46% or 18,188 of the patients missing in race. This is followed by admission type (7.84% or 10,745 missing records) and admission month (6.46% or 8,300 missing records).

**Table 1:** Characteristics of patients with total knee arthroplasty discharges in 2010–2014 NIS sample.

Variable	Frequency (n = 636,062)	Percent %
**Age group**		
44–64 years	269,508	42.34
65–74 years	230,683	36.28
75+ years	135,871	21.35
**Sex**		
Male	239,636	37.67
Female	396,426	62.33
**Race**		
White	479,689	75.44
Black	45,082	7.10
Hispanic	31,841	5.05
Asian or Pacific Islander	7,188	1.13
Native American	2,822	0.44
Other	13,258	2.09
Missing	56,182	8.75
**Median household income for patient’s zip code**		
0-25^th^ percentile	140,870	22.18
26^th^-50^th^ percentile	168,536	26.52
51^st^-75^th^ percentile	167,763	26.36
76^th^-100^th^ percentile	149,141	23.41
Missing	9,752	1.53
**Patient location**		
“Central” counties of metro areas ≥1 million pop	139,264	21.86
“Fringe” counties of metro areas ≥1 million pop	156,835	24.64
Counties in metro areas of 250,000–999,999 pop	134,036	21.15
Counties in metro areas of 50,000–249,999 pop	67,989	10.69
Micropolitan counties	81,815	12.84
Not metro/micropolitan counties	56,123	8.83
**Health insurance**		
Medicare	353,117	55.52
Medicaid	18,669	2.95
Private/HMO	241,355	37.94
Other	22,921	3.60
**Admission day**		
Admitted weekday	634,412	99.74
Admitted weekend	1650	0.26
**Admission type**		
Elective	113,712	85.62
Non-elective	8,568	6.54
Missing	10,745	7.84
**Admission month**		
January	55,852	8.78
February	49,048	7.71
March	50,891	8.00
April	49,740	7.83
May	47,694	7.50
June	52,656	8.28
July	48,116	7.58
August	48,795	7.67
September	49,947	7.66
October	59,312	9.34
November	54,575	8.59
December	49,692	7.82
Missing	19,744	3.05
**Bed size of hospital**		
Small	137,177	21.35
Medium	166,965	26.43
Large	331,920	52.22
**Location/teaching status of hospital**		
Rural	76,696	12.01
Urban non-teaching	274,241	43.04
Urban teaching	285,125	44.95
**Control/ownership of hospital**		
Government, non-federal	55,360	8.67
Private, not-for-profit	481,076	75.54
Private, investor-owned	99,626	15.80
**Year**		
2010	123,806	19.43
2011	126,540	19.34
2012	123,021	19.53
2013	129,552	20.56
2014	133,143	21.13
**Length of hospital stay**		
> = 3 days (prolonged)	468,946	73.63
< 3 days (normal)	167,116	26.37
**Discharge disposition**		
Routine discharge	171,329	26.96
Non-routine discharge	464,733	73.05
**Any complication**		
Yes	228,386	35.89
No	407,676	64.12
**Blood transfusion**		
Yes	73,020	11.41
No	563,042	88.59

Abbreviations: NIS, National (Nationwide) Inpatient Sample; pop, population; HMO, health maintenance organization.

### Disposition of patient at discharge

This outcome was defined as being either routine or non-routine in our study, and the models were generated to predict routine discharges. A majority of the patients (73.05%) were observed to have a non-routine discharge, implying that they required extra post-surgical care after the procedure was completed. Results from the three models are outlined in Table 2, where we observed that the GBM, RF and ANN models perform better than the LR model across all metrics for the test data. Specifically, the Brier score (where higher score indicates worse model) for the LR model was found to be 0.180—which was higher as compared to the scores of 0.132, 0.149 and 0.137 for the GBM, RF and ANN model (17–27% improvement achieved by ML models). The AUC (where higher score suggests better model) for the LR model was observed to be 0.685, which was lower than the measures for the GBM, RF and ANN models which were 0.857 (55% improvement), 0.841 (50% improvement) and 0.848 (52% improvement). It was observed that the F1-scores for routine discharges were lower for the LR model, with a score of 0.51, as compared to the GBM, RF and ANN models which had scores that were higher by 51–55%. The sensitivity score for the LR model (12%) was much lower than the ML models (≥64%). Additionally, all the ML models were observed to have high specificity (>80%), but the LR model had the comparatively highest specificity of 97%.

**Table 2:** Comparison of predictive model performance on test data using logistic regression and machine learning methods.

Outcome	Metrics	Logistic Regression	Gradient Boosting Machine	Random Forest	Neural Network
**Disposition of Patient at Discharge**	AUC AUC—95% CI	0.685 (0.682, 0.688)	0.857 (0.855, 0.859)	0.841 (0.838, 0.843)	0.848 (0.840, 0.856)
	Sensitivity	0.122	0.711	0.706	0.741
	Specificity	0.966	0.841	0.816	0.796
	F1 Score	0.201	0.664	0.641	0.647
	Brier Score	0.180	0.132	0.149	0.137
**Any Complication**	AUC AUC—95% CI	0.781 (0.779, 0.784)	0.871 (0.869, 0.872)	0.847 (0.845, 0.849)	0.861 (0.850, 0.871)
	Sensitivity	0.593	0.760	0.726	0.662
	Specificity	0.864	0.826	0.811	0.884
	F1 Score	0.646	0.734	0.704	0.707
	Brier Score	0.162	0.136	0.168	0.141
**Blood Transfusion**	AUC AUC—95% CI	0.707 (0.704, 0.711)	0.797 (0.794, 0.800)	0.783 (0.780, 0.787)	0.812 (0.805, 0.820)
	Sensitivity	0.450	0.531	0.517	0.525
	Specificity	0.817	0.862	0.854	0.865
	F1 Score	0.315	0.410	0.392	0.408
	Brier Score	0.095	0.091	0.094	0.088

Abbreviations: AUC, area under the receiver operating characteristic curve; CI, confidence interval; NIS, National Inpatient Sample.

### Any post-surgical complications

The predictions were modeled for the presence of any post-surgical outcomes. It was observed that more than half of the patients (64.12%) had no post-surgical complications. The results from the test data indicate that the GBM, RF and ANN models have better performance than the LR model (Table 2). The LR model had a higher Brier score (0.162) than the GBM and ANN model, which had scores of 0.136 and 0.141 (16% and 13% improvement achieved by the models, respectively). However, the Brier score of the RF model (0.168) was slightly higher than the LR model. The AUC for the LR model was found to be lower, with a value of 0.781, as compared to the GBM, RF and ANN models, which had AUC measures of 0.871 (11% improvement), 0.847 (8% improvement) and 0.861 (10% improvement). F1 scores are lower for the LR model as well, with a value of 0.646, as compared to the GBM, RF and ANN model which recorded improvements of 16–25%. The sensitivity score, and therefore the accuracy of predicting class 1 (post-surgical outcomes), was lower for the LR model (59%) as compared to the ML models (≥66%). All the predictive models were observed to have high specificity (>80%), and the ANN model had the highest specificity as compared to the other predictive models.

### Blood transfusion

The models were built to predict blood transfusion during TKA and predicted whether the patient had received an analogous blood transfusion during the course of their surgery. Frequency analyses revealed that a vast majority of the TKA patients (88.59%) did not receive blood transfusion. As noted in results from the test data in Table 2, the Brier score for the LR model (0.095) was marginally higher than the scores of 0.091 for the GBM, 0.094 for the RF and 0.088 for the ANN model (4%, 1% and 7% improvement, respectively). The LR model had a lower AUC (0.707) as compared to the AUC for the GBM (0.797), RF (0.783) and ANN (0.812) models, which showed an improvement of 31%, 26% and 36% respectively (Table 2). The LR model was also found to have a significantly lower F1-score of 0.315 as compared to 0.410 for the GBM (14% improvement), 0.392 for the RF (11% improvement) and 0.408 for the ANN (14% improvement) models. The sensitivity score of the LR model was 45%, which was lower than the scores observed for the ML models (51–53%). All the predictive models were observed to have good specificity, indicating a high degree of accuracy in predicting the outcome of not having received a blood transfusion, and the scores ranged from 81–87%. The LR model had comparatively lower specificity than the ML models.

### Summary of comparison measures

The performance measures used for evaluating the predictive models indicate that the ML models had better performance than the LR model for all the outcomes under consideration. Within the context of our study, the GBM model was found to have slightly better calibration and discrimination measures when compared to the RF and ANN based predictive models. On the basis of our results, we selected the GBM model as the representative ML algorithm in order to demonstrate the clinical applicability of the model. This was done by assessing the relative importance of the different predictor variables considered for each outcome of interest and subject-level predictions were also generated using the randomly sampled records from the study dataset. The code for our final model has been uploaded on GitHub for easy review and access by clinicians and other researchers (https://github.com/postincredible/TKA_predictive_modeling).

### Variable importance in the GBM model

**Fig 3:**
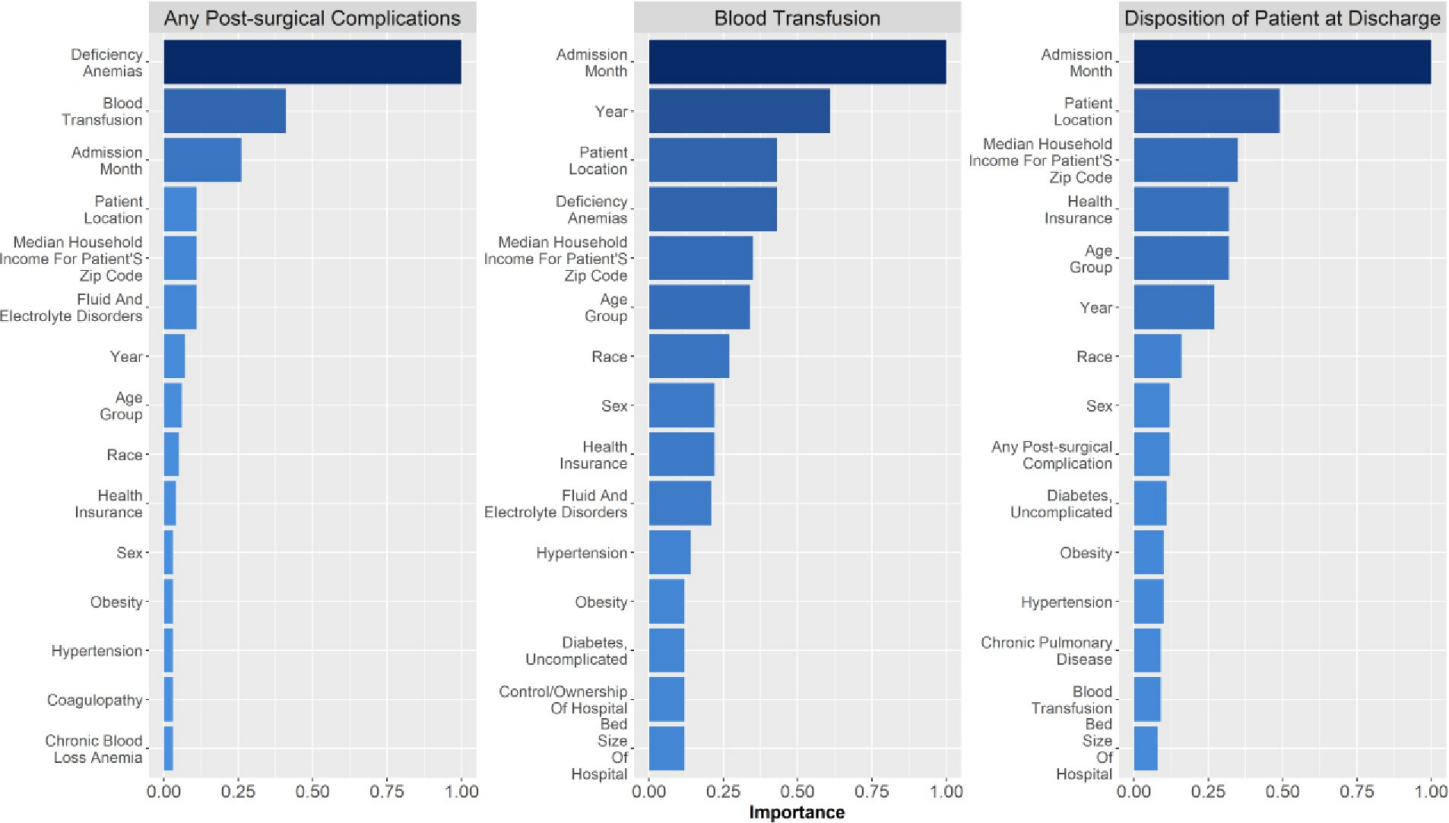
Relative importance of the predictor variables in the GBM predictive model.

Based on the predictive models, some individual variable importance graphs were generated for the different algorithms for the outcomes of interest. Specifically, findings from the GBM based predictive model have been reported due to relatively better performance of this model and Fig 3 is an illustrative example demonstrating the importance of the various predictor variables. For the outcome of any post-surgical complications, deficiency anemias, blood transfusion, admission month, patient location, and median household income for the patient were the predictors found to have the greatest significance in predicting the outcome. Blood transfusion was most significantly predicted by admission month, year of admission, patient location, deficiency anemia as a comorbidity, and median household income for the patient. The strongest predictors for the outcome of discharge disposition were observed to be admission month, patient location, median household income for the patient, health insurance provider and age category. A similar illustrative figure has also been generated for the RF model (Appendix, S4 Fig).

### Patient-level predictions using the GBM model

**Fig 4:**
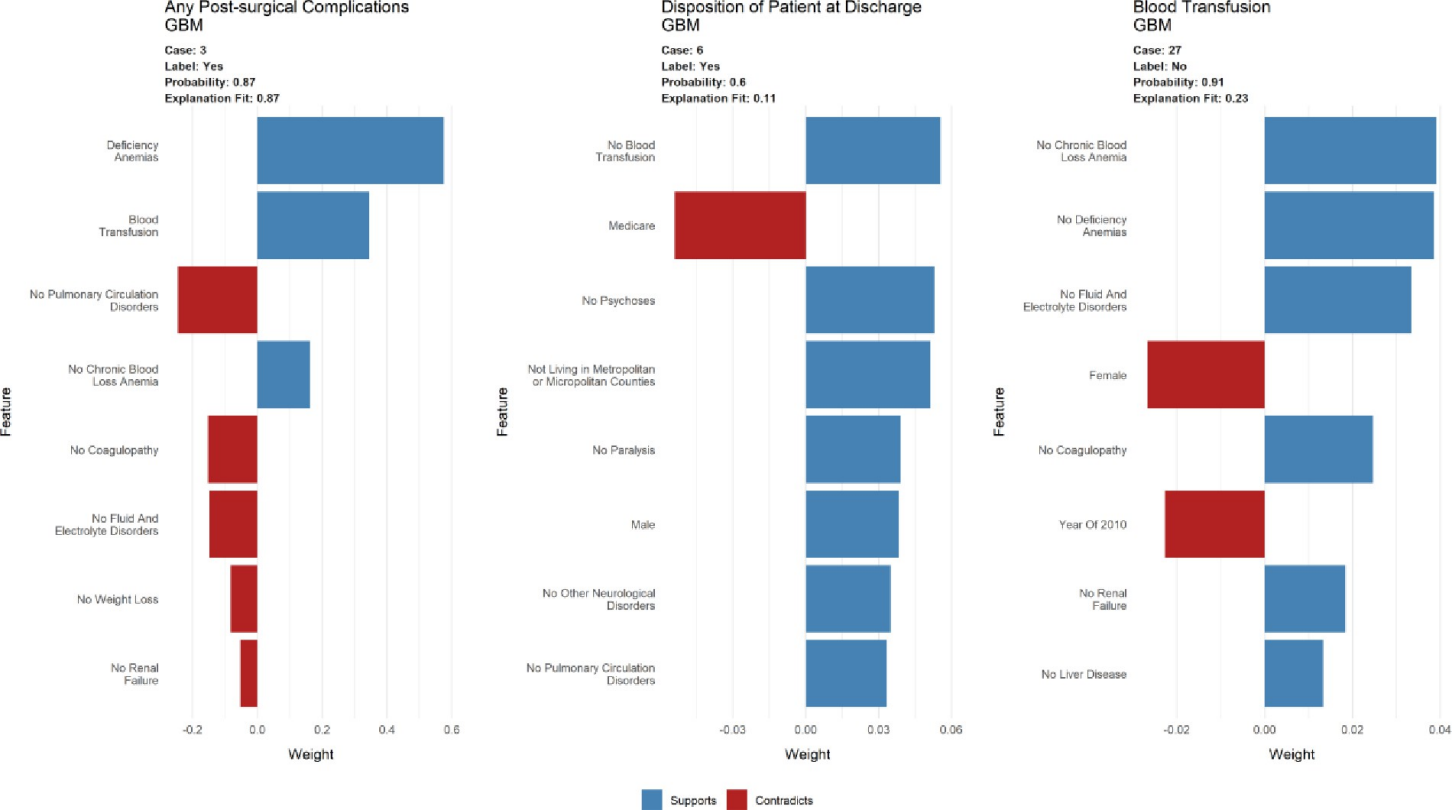
Diagrammatic representation of the patient-level predictions obtained using the GBM predictive models.

Since these predictive models have been built to ultimately predict post-surgical outcomes for individual patients, we generated variable importance graphs for patient-level outcomes in order to assess the importance of different patient- and hospital-level predictor variables. Fig 4 provides an illustrative example using the GBM model, since this prediction algorithm was observed to have better overall performance as compared to the other methods assessed in this paper. For the outcome of any post-surgical outcomes, case 3 from the dataset was used as a test subject in order to identify the most significant predictors for this patient’s clinical outcome. The patient was observed to have a high probability (87%) of having any post-surgical complications following their TKA procedure. The presence of the anemia deficiency comorbidity, absence of blood loss comorbidity, and blood transfusion conducted after the procedure were found to positively predict the outcome. On the other hand, the absence of the comorbidities of pulmonary circulation disorders, coagulopathy, fluid and electrolyte disorders, weight loss, and renal failure were negatively associated with the probability of the patient suffering from any post-surgical complications. Fig 4 also contains similar graphs outlining the direction of association of the most significant predictor variables for the other two outcomes under consideration–disposition of patient at discharge and blood transfusion.

## Discussion

The models built in this study are used to predict the clinically relevant outcomes of discharge disposition, post-surgical complications and blood transfusion procedures among 636,062 patients in the NIS database that had undergone TKA between 2010 to 2014. ML-based models performed better in this capacity to predict post-surgical outcomes, and these models were found to have consistently better calibration and discrimination measures as compared to the LR models. Within the ML models, the GBM model was observed to have slightly better overall performance and the assessment of the relative importance of the predictor variables used in this model revealed some significant common predictors for the three adverse outcomes under investigation. In order to demonstrate the clinical applicability of this ML model, patient-level predictions were also generated using sample records from the NIS database for the outcomes under investigation in order to better understand the impact of the predictor variables that could help physicians identify the patient’s likelihood of experiencing complications following their surgical procedure.

The predictors used in the GBM model which were observed to have the most importance in predicting the disposition of patients at discharge were as follows–admission month, patient location, median household income for patient’s zip code, health insurance, age group, year, race, sex, any post-surgical complications, diabetes (uncomplicated), obesity, hypertension, chronic pulmonary diseases, blood transfusion, and bed size of hospital. Studies that have previously been conducted in order to predict discharge disposition following orthopedic surgeries have reported some of the same predictors which were observed to have the highest degree of significance in the predictive model. Barsoum et al. had observed that discharge disposition following total joint arthroplasty (TJA) was also significantly predicted by age, gender, diabetes, hypertension, pulmonary conditions, infection, and home location for the patient [22]. Lu et al. had developed predictive models using extreme gradient boosting methods in order to assess the probability of nonroutine discharge following unicompartmental knee arthroplasty, and they had also noted that length of stay, age, gender, and diabetes were significant predictors for this adverse outcome [23]. Based on our literature review, we identified the following unique significant predictors during our analyses for the outcome of discharge disposition–admission month, year, health insurance provider, race, obesity, and any post-surgical complications. The variable for any post-surgical complications includes diagnosis codes for a wide range of common complications (Table II, Appendix), and clinicians could potentially benefit from taking these various complications under consideration while assessing the probability of the patient requiring extended care upon discharge. Predictors such as race and health insurance status are important proxy indicators for socioeconomic status, and provide an avenue for future research in order to better understand the inequities in healthcare outcomes such as the patient disposition after discharge can be affected by these variables.

For the outcome of blood transfusion, the most significant predictors in the GBM model were–admission month, year, patient location, deficiency anemias, median household income for patient’s zip code, age group, race, sex, health insurance, fluid and electrolyte disorders, hypertension, obesity, diabetes (uncomplicated), control/ownership of hospital, and hospital bed size. Frisch et al. have previously developed predictive models for predicting blood transfusion following total hip and knee arthroplasty and the common significant predictors that they had noted are as follows–gender, age, and BMI (indicator for obesity) [24]. Yoshihara and Yoneoka had also assessed the significant predictors for allogenic blood transfusion (ALBT) following total hip and knee arthroplasty, and reported the following similar significant predictors for ALBT after TKA–age group, gender, anemia, insurance status, and hospital ownership [25]. In our study, we found that the significant predictor variables for admission month, patient location, patient income, race, fluid and electrolyte disorders, hypertension, diabetes, and hospital bed size had previously not been reported to be important indicators for the outcome of blood transfusion among patients undergoing TKA, based on our review of existing literature. Similar to the outcome of discharge disposition, we observed that certain representative indicators of the socioeconomic status of the patient–patient location, patient income, and race–play an important role in the prediction of this adverse outcome. Taking these variables into consideration while designing treatment plans can help improve patient outcomes following TKA and other orthopedic procedures in a more equitable manner. The other unique variables identified included certain patient comorbidities, and further clinical research can help identify the biological mechanisms that cause patients with these comorbidities to require blood transfusions following TKA.

The outcome variable for any post-surgical complications was generated using specific diagnosis codes for this study, and there are no other studies for comparing the reported significant predictors. The most significant predictors for this outcome were observed to be deficiency anemias, blood transfusion, admission month, patient location, median household income for patient’s zip code, fluid and electrolyte disorders, year, age group, race, health insurance, sex, obesity, hypertension, coagulopathy, and chronic blood loss anemia. These results indicate that comorbid conditions are likely to put the patient at a high risk of suffering from various complications following TKA. Comorbidities that could cause substantial loss of blood are especially relevant in predicting this outcome. In accordance with our previous observations regarding the patient’s discharge disposition and blood transfusion following TKA, proxy indicators for socioeconomic status–such as race and health insurance–play a significant role in predicting any post-surgical complications as well. The results from all of the three outcomes under investigation generally indicate that there are some common risk factors for the adverse outcomes that can be applicable across different cohorts of patients, and can therefore be accounted for during the process of designing the treatment plan for any patient who is scheduled to undergo an orthopedic surgical procedure.

Using the GBM model, we also generated some patient-level predictions for the outcomes under investigation. Patient records from the study dataset were used for these models and we were able to successfully demonstrate the potential real-world utility of using the predictive model as a tool for aiding in the decision-making process prior to conducting a TKA procedure. Significant predictors, and their corresponding weightage in terms of their relevance to the adverse outcome under investigation, can be generated for individual patients using the final GBM model. Similar models could also be generated for other orthopedic surgical procedures in order to assess specific outcomes for the patients on the basis of their clinical importance.

One of the primary strengths of our study is making use of this large nationally representative healthcare dataset for training the predictive models, so that they can learn complex and meaningful patterns not usually available in other datasets. However, the NIS database consists of a moderate amount of missing data for a number of demographic variables which can, in turn, affect the accuracy of predictive models being generated [26]. This is because ignoring missing data would introduce a certain degree of bias into the study results by affecting the observed relationships between the predictor and outcome variables [27]. By conducting SRMI in our study, we negate this effect of missing data on the study results and increase the overall performance of the predictive models. Another important consideration while building predictive models is to minimize the risk of overfitting. Overfitting is a common issue among predictive models and occurs when the model tracks and “memorizes” the patterns that exist in the training data, limiting their predictive abilities and inhibiting their generalization to the test and other clinical datasets [28]. It can be identified by comparing the model performance between the training and test datasets, and determining whether the model performance in the test data is significantly worse than the training data. Our results indicate that there was minimal overfitting in any of the predictive models.

One of the limitations of our study is that the final GBM based predictive model was not externally validated using a separate dataset. External validation should ideally be conducted using other large datasets (eg. Medicare or Medicaid data) so that the predictive power of the final model can be demonstrated across different datasets; this was beyond the scope of this study, but will be explored further in future research. Moreover, sufficient comparisons could not be made between the three ML models and they had been observed to have very similar performance, in terms of calibration and discrimination. This is partially because fine-tuning the hyperparameters was not conducted extensively for the models during the course of this study. The performance of ML models depends on how they are individually fine-tuned, which is complicated enough to warrant a thorough investigation. Work is underway to compare the performance of different ML models for prediction of surgical outcomes.

An additional limitation of this work is that we only performed feature selection for Logistic Regression based on backward selection but not for the three Machine Learning models. However, as the Machine Learning models already outperformed Logistic Regression, had we conducted feature selection for these models their performance would have been even better than that of Logistic Regression. In other words, our reported findings will only be strengthened, rather than weakened, by including feature selection. In our future work we plan to perform extensive fine-tuning of our pipeline. Concretely, we will fine-tune data preprocessing including conducting feature selection, feature engineering, dimensionality reduction. We will also fine-tune the key hyperparameters of each model. The best setting of the fine-tuning will be reported so as to provide good practices to researchers.

## Conclusions

We achieved our objective of developing predictive models based on LR and ML-based methods, and further demonstrated the importance of previously unreported proxy indicators for socioeconomic factors that can help predict the likelihood of patients experiencing adverse outcomes after the TKA procedure. The final predictive model recommended for clinical applications based on the comparative analysis conducted in our study was the GBM model. In summary, ML-based methods have significant potential for their applicability in determining risk factors for patient cohorts undergoing orthopedic and other surgical procedures, and should be highly recommended since they are readily available in major statistical software. Prior knowledge of these factors can aid clinicians in making decisions with the most optimal outcomes from the lens of both economic viability and patient-centered healthcare.

## Supporting information

S1 Fig Demonstration of simple gradient boosting decision trees. (TIF)

S2 Fig An illustrative simple decision tree structure. (TIF)

S3 Fig An illustrative 3-layer ANN structure. (TIF)

S4 Fig Diagrammatic representation of the patient-level predictions obtained using the RF predictive model. (TIF)

S1 File Additional tables and supplemental information. (DOCX)
