# Supplementary figures and images for: Utilization of machine learning methods for predicting surgical outcomes after total knee arthroplasty
Source: PLoS One. 2022 Mar 22;17(3):e0263897. doi: 10.1371/journal.pone.0263897 (PMC8939835; doi:10.1371/journal.pone.0263897)

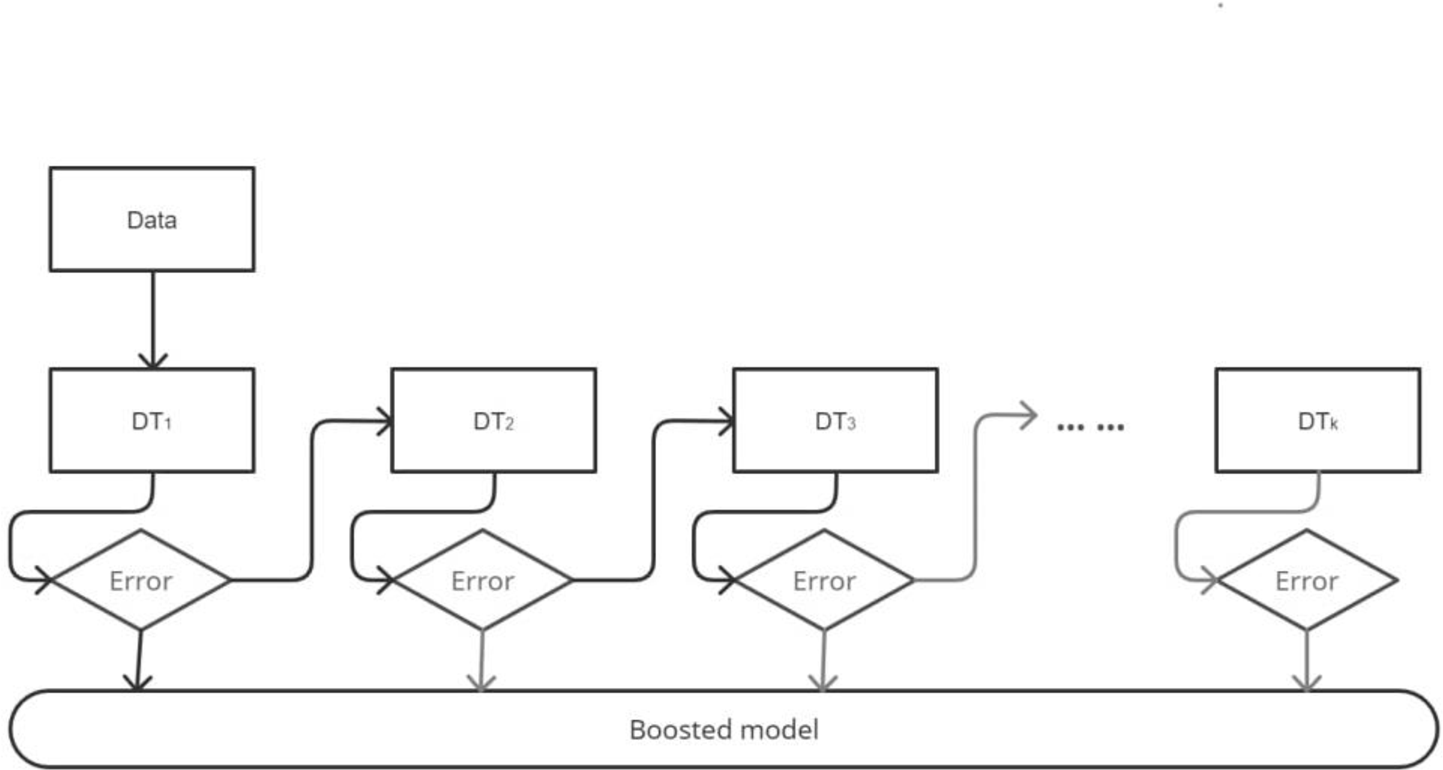

Supplement: S1 Fig — (TIF) [file pone.0263897.s001.tif]

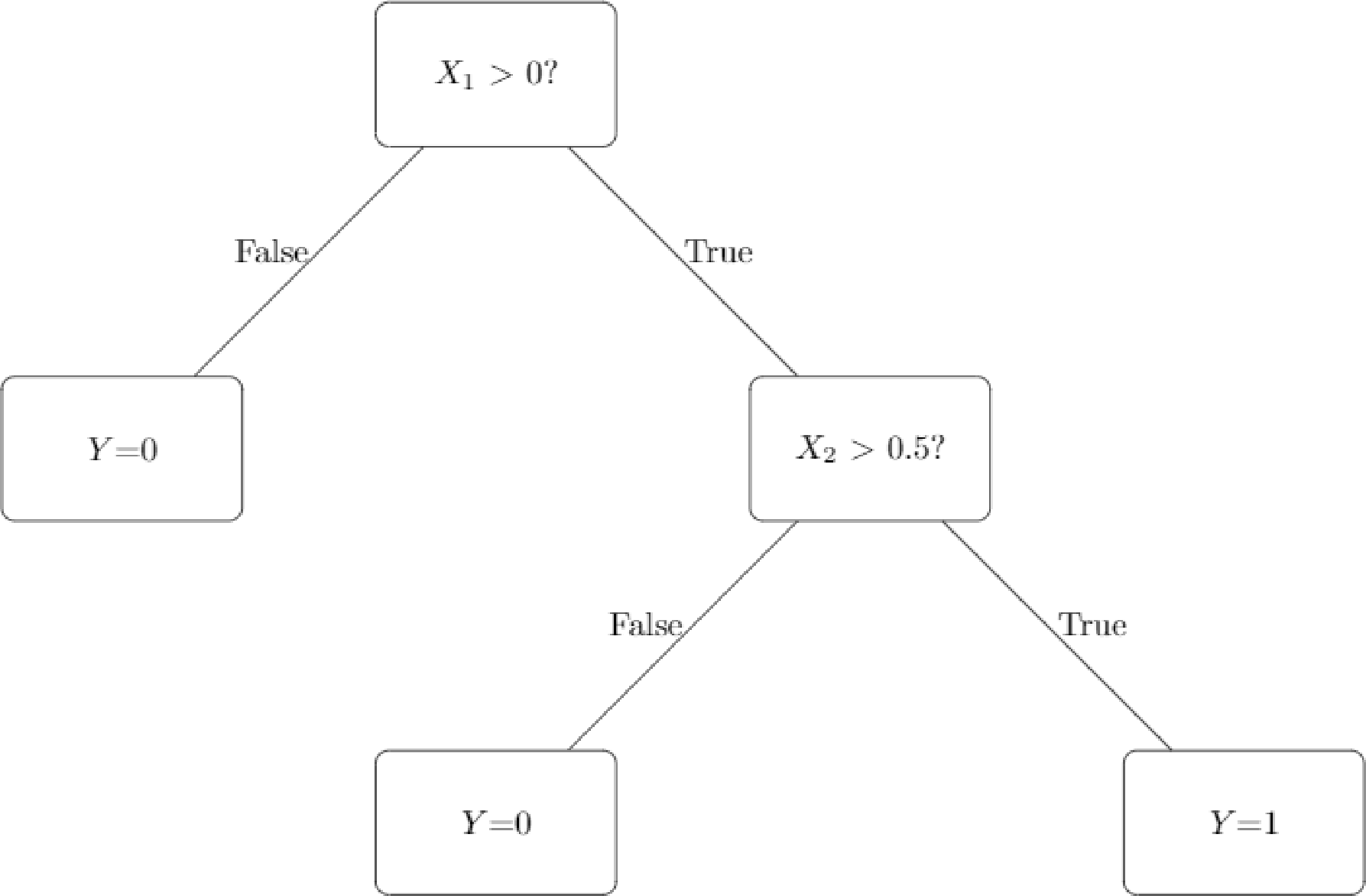

Supplement: S2 Fig — (TIF) [file pone.0263897.s002.tif]

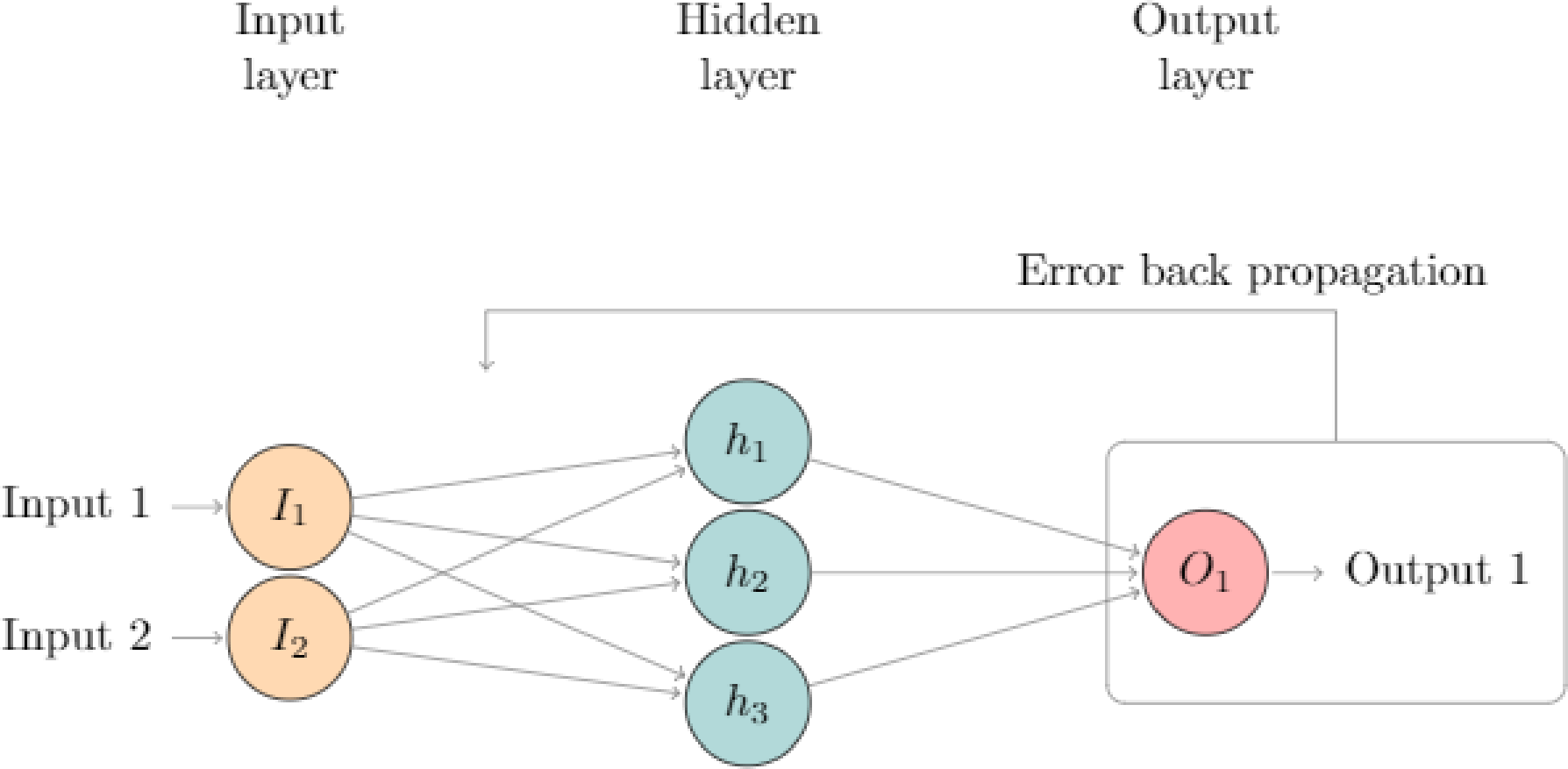

Supplement: S3 Fig — (TIF) [file pone.0263897.s003.tif]

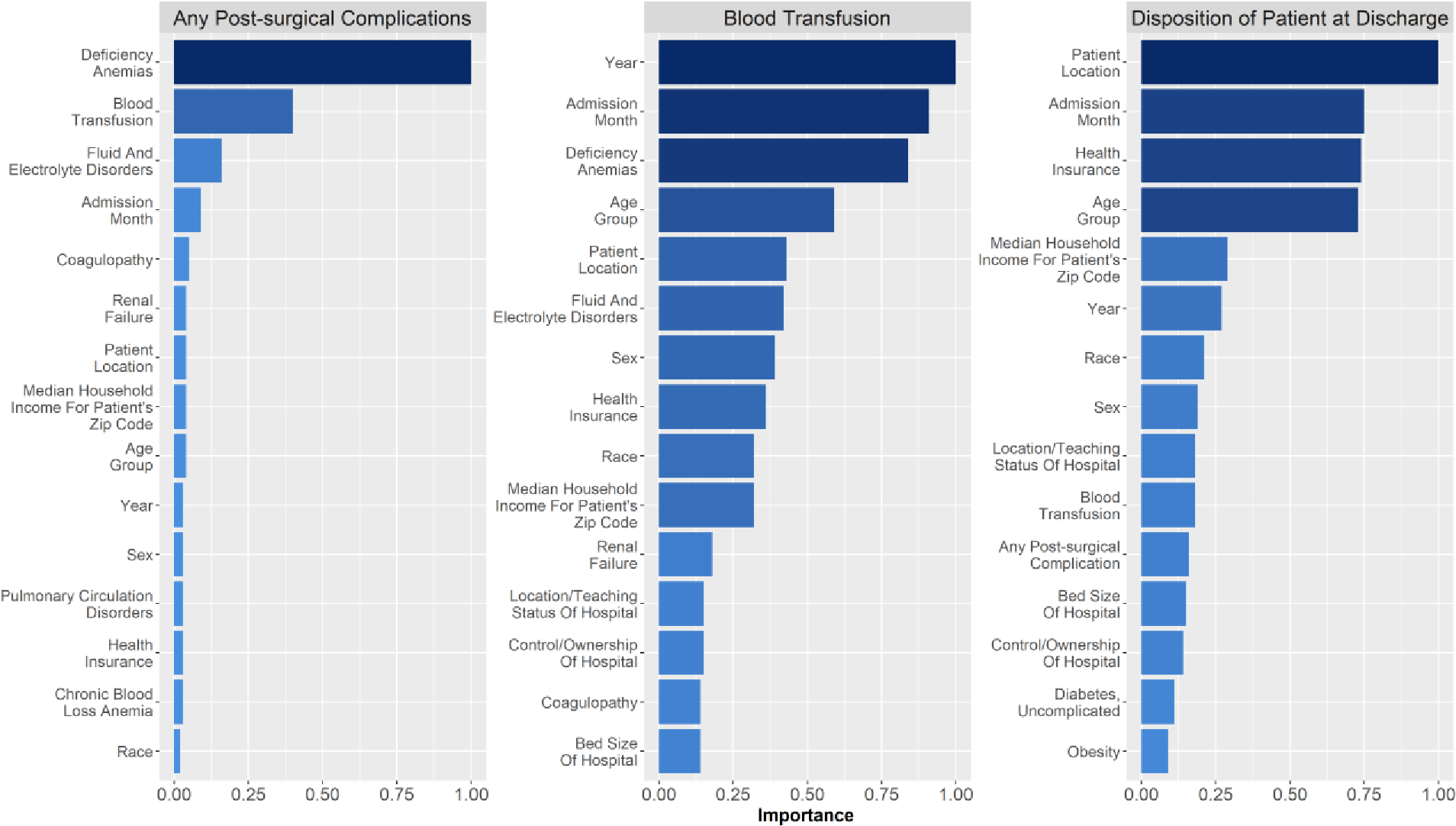

Supplement: S4 Fig — (TIF) [file pone.0263897.s004.tif]
