# Supplementary material for: Utilization of machine learning methods for predicting surgical outcomes after total knee arthroplasty
Source: PLoS One. 2022 Mar 22;17(3):e0263897. doi: 10.1371/journal.pone.0263897 (PMC8939835; doi:10.1371/journal.pone.0263897)
Supplement: S1 File — (DOCX) [file pone.0263897.s005.docx]

**Manuscript Title:** Utilization of Machine Learning Methods for Predicting Surgical Outcomes after Total Knee Arthroplasty

**APPENDIX**

| Table I. Description of ICD-9 procedure codes used for generating the outcome variable of blood transfusion | |
| --- | --- |
| **ICD-9 code** | **Category of blood transfusion** |
| **99.00, 99.02** | Autologous blood transfusion |
| **99.03, 99.04** | Allogenic blood transfusion |

| Table II. Description of ICD-9 diagnosis codes used for generating the outcome variable of any post-surgical complications | |
| --- | --- |
| **ICD-9 code** | **Category of post-surgical complications** |
| **996.xx*** | **Complications peculiar to certain specified procedures**  (Includes: Mechanical complications associated with implanted devices and grafts; Infections due to internal prosthetic device, implant and grafts; and Complications of transplanted organs and reattached body parts) |
| **997.xx*** | **Complications affecting specified body systems, not elsewhere classified**  (Includes: Nervous system complications; Respiratory complications; Digestive system complications; Amputation complications; Vascular complications; and Complications in specified body systems, not elsewhere classified) |
| **285.xx*** | **Other and unspecified anemias**  (Includes: Sideroblastic anemia; Post-hemorrhagic anemia; and Anemia associated with chronic diseases) |
| **415.xx*** | **Acute pulmonary heart disease**  (Includes: Acute cor pulmonale; Pulmonary embolism and infarction; and Phlebitis and thrombophlebitis at various sites) |
| **453.xx*** | **Other venous embolism and thrombosis**  (Includes: Budd-chiari syndrome; Thrombophlebitis migrans; Embolism at various sites; and Chronic venous embolism at various sites) |
| **518.xx*** | **Other diseases of lung**  (Includes: Pulmonary collapse; Emphysema; Edema in the lungs; Pulmonary insufficiency following trauma and surgery; Allergic bronchopulmonary aspergilliosis; Transfusion related lung injury; and Other diseases of lung) |
| *where “x” denotes an integer between 0-9 | |

| Table III**.** Comparison of predictive model performance on training data using logistic regression and machine learning methods | | | | | |
| --- | --- | --- | --- | --- | --- |
| **Outcome** | **Metrics** | **Logistic Regression** | **Gradient Boosting Machine** | **Random Forest** | **Neural Network** |
| **Disposition of Patient at Discharge** | AUC  AUC - 95% CI | 0.685  (0.683, 0.686) | 0.947  (0.946, 0.947) | 0.919  (0.917, 0.921) | 0.856  (0.846, 0.865) |
|  | Sensitivity  Specificity  F1 Score  Brier Score | 0.123  0.966  0.202  0.180 | 0.881  0.887  0.806  0.091 | 0.841  0.847  0.746  0.128 | 0.749  0.801  0.654  0.134 |
| **Any Complication** | AUC  AUC - 95% CI | 0.783  (0.781, 0.784) | 0.933  (0.930, 0.936) | 0.901  (0.900, 0.902) | 0.870  (0.857, 0.883) |
|  | Sensitivity  Specificity  F1 Score  Brier Score | 0.595  0.864  0.648  0.161 | 0.840  0.874  0.813  0.107 | 0.780  0.854  0.765  0.152 | 0.673  0.887  0.716  0.138 |
| **Blood Transfusion** | AUC  AUC - 95% CI | 0.709  (0.706, 0.711) | 0.907  (0.826, 0.988) | 0.898  (0.897, 0.900) | 0.834  (0.827, 0.840) |
|  | Sensitivity  Specificity  F1 Score  Brier Score | 0.452  0.817  0.315  0.095 | 0.768  0.883  0.575  0.073 | 0.746  0.874  0.549  0.084 | 0.551  0.867  0.425  0.085 |

Abbreviations: AUC, area under the receiver operating characteristic curve; CI, confidence interval; NIS, National Inpatient Sample.

***Model Description***

*Gradient Boosting Machine*

GBM is usually referred to as gradient boosting decision trees, which is an extremely powerful and popular machine learning (ML) algorithm in regression and classification. Instead of combining independent trees as in RF, GBMs combine individual decision trees through a boosting method [1]. In this method, decision trees are built in a sequential manner and each tree is built based on the error from its previous tree and the final prediction will be the sum of the predictions from all trees (Appendix, Figure I). As each tree in GBM is designed to minimize the error of its previous tree, one can use many weak learners (shallow trees) to make a boosted model with good predictive power. The GBM models were built using the *h2o* package in R [2-4].

*Random Forest Model*

RF is a classification model consisting of a number of individual decision trees (DTs). A DT is the fundamental structure of RF [5, 6]. Figure 2 in the Appendix shows a simple DT structure for a binary outcome Y and two predictors X1 and X2. We predict Y as 0 when X1 <= 0, or when X1 > 0 and X2 <= 0.5. Conversely, we predict Y as 1 when X1 > 0 and X2 > 0.5. The fact that the DT uses the interaction between X1 and X2 to predict Y demonstrates the ability of the model to automatically capture complex relationships between the predictors and outcome. The final prediction of RF is then decided by the majority vote across the DTs. For example, consider a RF with 3 DTs. If two trees predict Y as 1 and one tree predicts Y as 0, then the final prediction of RF is Y=1. The RF models were built using the *h2o* package in R [2-4].

*Artificial Neural Network Model*

ANN is another powerful model that can automatically capture the complex relationships between predictors and outcome [7]. Our study used a feedforward fully connected ANN, which consists of an input layer, multiple hidden layers, and an output layer. Figure 3 in the Appendix illustrates a hypothetical ANN model with a single hidden layer. Within a layer, there are several nodes, and each node is fully connected with nodes in its adjacent layers. Nodes in the input layer represent predictors. A set of nodes at the hidden layer represents mathematical functions that modify the input information and improve prediction. The output layer collects the predictions made in the hidden layer and produces the final prediction. The ANN models were built using the *h2o* package in R [2-4].

***Model Parameters***

All parameters in machine learning (ML) methods are obtained from a random search of predefined parameter space with an AUC-based early stopping rule. Detailed settings in ML models can be found in the GitHub sample code (<https://github.com/postincredible/TKA_predictive_modeling>).

List of parameters space for each ML method:

1. RF

max_depth = c(3, 5, 10, 15),
sample_rate = c(0.8, 1.0),
col_sample_rate_change_per_level = c(0.9, 1, 1.1),
col_sample_rate_per_tree = c(0.5, 0.8, 1.0),
ntrees=c(50, 100, 200)

1. GBM

max_depth = c(3, 5, 10, 15),
sample_rate = c(0.8, 1.0),
col_sample_rate = c(0.5, 0.8, 1),
col_sample_rate_change_per_level = seq(0.9, 1, 1.1),
col_sample_rate_per_tree = c(0.5, 0.8, 1.0),
ntrees=c(50, 100, 200))

1. ANN

hidden=list(c(200,200),c(320,400,80),c(125,125,125,125)),
epochs = c(50, 100, 200),
l1 = c(0, 0.00001, 0.0001),
l2 = c(0, 0.00001, 0.0001),
rho = c(0.9, 0.95, 0.99, 0.999),
epsilon = c(1e-10, 1e-8, 1e-6, 1e-4),
input_dropout_ratio = c(0, 0.1, 0.2),
max_w2 = c(10, 100, 1000, 3.4028235e+38)

**REFERENCES**

1. Hastie T, Tibshirani R, Friedman J. The elements of statistical learning: data mining, inference, and prediction. Springer Science \& Business Media; 2009.
2. R Core Team. R: A language and environment for statistical computing. R Foundation for Statistical Computing, Vienna, Austria; 2020. URL <https://www.R-project.org/>.
3. Cook D. Practical Machine Learning with H2O: Powerful, Scalable Techniques for AI and Deep Learning. O’Reilly Media, Inc.; 2016.
4. Aiello S, Eckstrand E, Fu A, Landry M, Aboyoun P. Machine Learning with R and H2O. H2O Bookl. 2016;550.
5. Breiman L. Random forests. Machine learning. 2001;45(1):5-32.
6. Shi T, Horvath S. Unsupervised Learning With Random Forest Predictors. J Comput Graph Stat [Internet]. 2006 Mar;15(1):118–38. Available from: https://www.tandfonline.com/doi/full/10.1198/106186006X94072
7. Yegnanarayana B. 2009. Artificial Neural Networks. PHI Learning. Available from: https://books.google.com/books?id=RTtvUVU_xL4C.
